# Immunological Evaluation of PLLA/GO Scaffolds in Goat Mandibular Bone Regeneration

**DOI:** 10.1002/mabi.202500362

**Published:** 2025-12-05

**Authors:** Thamires Santos‐Silva, Fernando Gonçalves da Silva Petrônio, Inácio Silva Viana, Raí André Querino Candelaria, Ana Caroline dos Santos, Paulo Alescio Canola, Luís Gustavo Gosuen G. Dias, Rodrigo da Silva Nunes Barreto, Michel Matar, Marcelo Melo Soares, Maria Angelica Miglino

**Affiliations:** ^1^ Department of Surgery School of Veterinary Medicine and Animal Science University of São Paulo São Paulo Brazil; ^2^ Department of Animal Anatomy University of Marilia Marília São Paulo Brazil; ^3^ Department of Veterinary Clinic and Surgery School of Agricultural and Veterinary Sciences São Paulo State University Jaboticabal São Paulo Brazil; ^4^ Department of Animal Morphology and Physiology Faculty of Agricultural and Veterinary Sciences São Paulo State University Jaboticabal São Paulo Brazil; ^5^ Institute of Orofacial Osteogenesis Rehabilitation S/S Ltda São Paulo Brazil

**Keywords:** bone neoformation, composites, goat mandible, immunology, regenerative medicine

## Abstract

Bone repair remains a significant challenge in orthopedics and reconstructive surgery, especially in complex fractures and large tissue defects. Biomaterials such as graphene oxide (GO) and poly‐L‐lactic acid (PLLA) are promising due to their physicochemical properties and biocompatibility. However, the immune response plays a critical role in graft success. In this study, PLLA/GO scaffolds are implanted in the right antimeres of nine goat mandibles, while the left antimeres are stabilized with titanium plates and serve as controls. Samples are collected at 15‐, 45‐, and 60‐days post‐ implantation and examined using histology and immunohistochemistry to assess inflammation, bone organization, and bone–implant integration. At 15 days, hematoxylin and eosin staining show early inflammatory reactions at the PLLA/GO interface, whereas control samples display regular histological patterns. At 45 days, denser and more compact bone tissue indicate progressive structural organization. By 60 days, the samples present advanced bone maturation and active osteogenesis. Steven's Blue staining confirms mineral deposition, and osteoblast‐like cells deposit new matrix at the scaffold–tissue interface. Immunohistochemical detection of osteocalcin and VEGF reveals osteoblastic activity and angiogenesis. These results demonstrate that PLLA/GO scaffolds promote bone regeneration and integration, supporting their potential for clinical application in mandibular repair.

## Graphical abstract

PLLA/GO scaffolds interact with bone tissue by modulating the immune response and promoting regeneration in goat mandibles. Histological and immunohistochemical analyses reveal progressive tissue organization, mineral deposition, osteoblastic activity, and angiogenesis over time. These findings highlight the clinical potential of this biomaterial for repairing complex fractures and large bone defects.

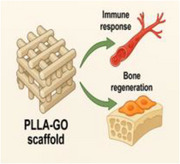

## 1 Introduction

Bone repair remains a challenge in orthopedic medicine and reconstructive surgery [1, 2, 3]. Biomaterials have emerged as a promising approach to stimulate bone regeneration in complex fractures and extensive bone loss. Among these biomaterials, graphene oxide (GO) and poly‐L‐lactic acid (PLLA) have gained attention due to their favorable physicochemical properties and ability to support cellular and tissue growth. These materials are also widely studied and thoroughly characterized [4, 5, 6, 7].

However, the successful transplantation of these biomaterials heavily depends on the host immune response, which can vary considerably [8, 9, 10]. The interaction between the immune system and biomaterials is complex and directly affects the effectiveness of bone repair and implant integration with surrounding tissue [9, 11, 12, 13]. The variability in individual immune responses, combined with the biomaterial ability to modulate this response favorably, poses a major challenge in optimizing clinical outcomes [9, 14, 15]. Thus, understanding the immunological mechanisms involved in bone repair is fundamental for optimizing the development and clinical application of these materials [16, 17, 18].

A key mechanism in tissue repair is the presence of inflammatory cells, which trigger essential events for the proper regeneration of damaged tissue [19, 20]. In the initial phase of bone repair, macrophages and neutrophils remove cellular debris and microorganisms [21]. Macrophages, in particular, release growth factors such as platelet‐derived growth factor (PDGF), transforming growth factor‐beta (TGF‐β), basic fibroblast growth factor (bFGF), and cytokines to promote cell proliferation and angiogenesis [22, 23, 24]. These factors also contribute to the formation of the initial bone callus. However, prolonged or dysregulated inflammation can lead to adverse effects, such as excessive formation of fibrous tissue instead of bone, thus compromising the structural and functional integrity of the repairing bone [25].

Bone repair is a multifactorial process that requires precise coordination of cellular and molecular events. Immunohistochemical analysis plays a crucial role in this context by enabling the visualization and quantification of specific proteins and inflammatory cells in the tissue. This technique provides valuable insights into cellular dynamics during healing and is key to understanding the mechanisms underlying bone regeneration, as well as identifying potential therapeutic targets [26, 27].

Given the importance of understanding these mechanisms for developing therapies that optimize bone healing in various clinical conditions, this study investigates the immune response to PLLA/GO scaffolds in a goat model for bone repair. By analyzing the biological processes involved in tissue regeneration, we aim to deepen our understanding of how these biomaterials influence bone healing.

## 2 Results and Discussion

### 2.1 Microscopic Analysis

Bone tissue is a specialized type of connective tissue composed of cells and a calcified extracellular matrix, i.e., the bone matrix [28, 29]. This matrix consists of a mineral component (calcium phosphate) and an organic component (95% type I collagen, glycosaminoglycans, proteoglycans, and glycoproteins such as osteonectin) [30]. Histologically, bones are classified as either primary (immature) or secondary (mature or lamellar), with the latter appearing in various stages of bone modeling and remodeling [31, 32].

**FIGURE 1:**
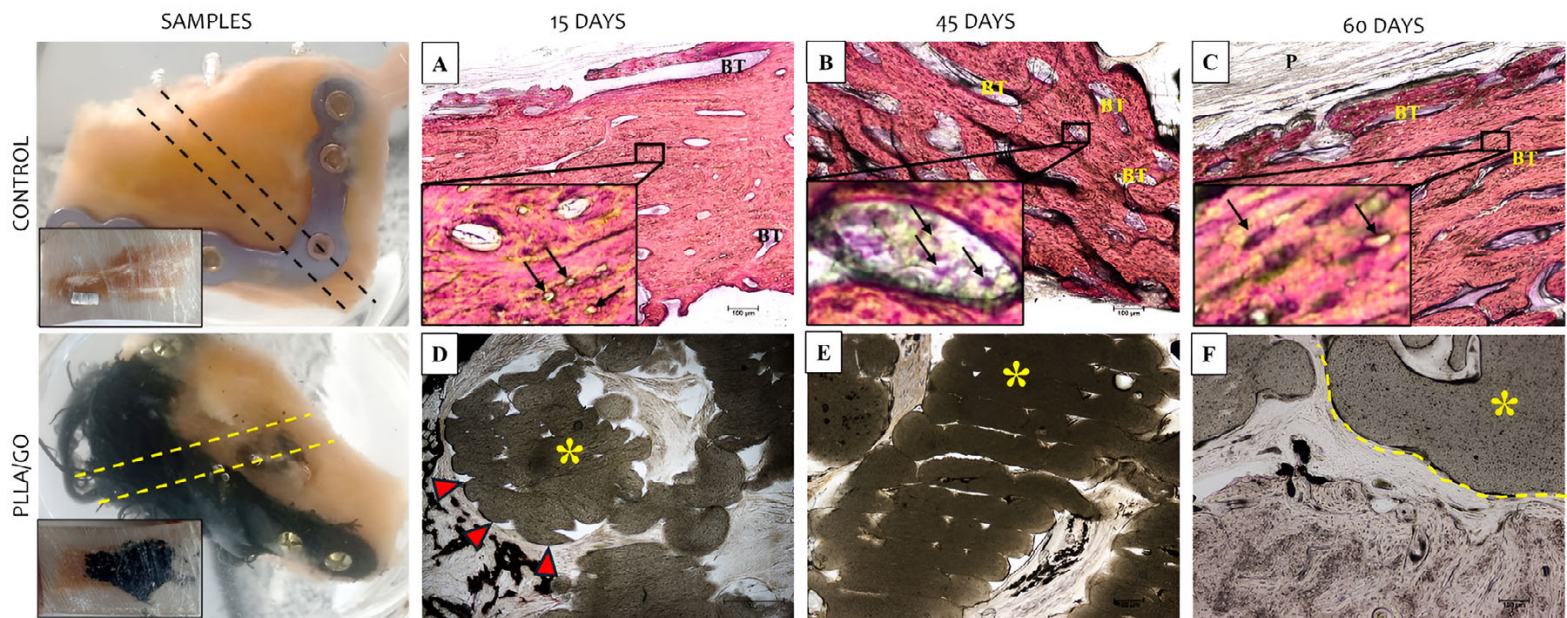
Histological images (HE staining) of goat mandibles from control (A–C) and PLLA/GO scaffold‐treated groups (D–F) at 15, 45, and 60 days. Bone matrix (pink), osteocytes (black arrows), bone trabeculae (BT), periosteum (P), and scaffold regions (*) are shown. The bone–implant interface is indicated by red arrowheads (inflammatory response) and a yellow dashed line. Scale bar: 100 µm.

Figure 1 shows H&E‐stained histological sections of the control implants (titanium plate) and PLLA/GO scaffold at 15, 45, and 60 days. The control groups exhibited a consistent pattern across all animals. The bone matrix, stained pink, showed a high collagen content between the lacunae (small cavities). Beneath this bone matrix, a large volume of bone cells was observed, such as osteocytes (black dots) centrally located within the lacunae. The periosteum (the fibrous layer covering the bone) was visible at the outermost layer. Moreover, the bone trabeculae appeared as a denser and more continuous band of tissue, organized into elongated and branched patterns with interspersed lacunae. These findings indicate that the titanium plate maintained normal cell activity (Figure 1A–C).

Liu et al. [33] conducted an *in vitro* study using porous nickel‐titanium (NiTi) implants, which resulted in cell growth on the material surface after eight days of culture. They found that surface roughness and NiTi topography significantly influenced cell adhesion and proliferation. In contrast, Ding et al. [34] investigated bone repair and osseointegration using titanium implants and found that the materials in their study failed to form a strong bond with the surrounding tissue after implantation. This weak interaction could lead to implant loosening or displacement due to low surface activity.

In the group that received PLLA/GO scaffold implants at 15 days, the implant material was clearly identifiable as darker regions in the images, whereas the lighter surrounding areas showed the adjacent bone tissue and its cells. The PLLA/GO implant appeared as a dense, dark mass with well‐defined margins relative to the surrounding tissue. The interface between the implant and bone did not exhibit clear signs of integration, with evidence of an initial inflammatory response (lighter areas). This response may indicate the presence of macrophages or multinucleated giant cells, typical of a foreign body reaction, suggesting that the implant material triggered a localized immune response [35]. The presence of these cells suggests that the body was attempting to isolate or degrade the implant material as part of an adaptation or rejection process.

The bone tissue surrounding the implant was visible, with osteocytes lodged in lacunae and distributed throughout the matrix. These osteocytes are mature cells derived from osteoblasts and play an important role in maintaining the bone matrix. In certain areas, the bone matrix appeared denser, reflecting its trabecular structure. Shayesteh et al. [36] and Hart et al. [31] reported that irregularly arranged collagen fibers without a defined orientation are typical of immature bone tissue. Thus, this finding was expected, as after an injury (critical defect), a rapid, protective, and restorative response occurred to repair the damaged or weakened tissue (Figure 1D).

In the 45‐day group, the surrounding bone tissue appeared denser and more compact, suggesting advanced tissue organization. The bone trabeculae were thicker and appeared more mature, reflecting an advanced stage of osteogenesis. Compared to the initial 15‐day stage, in which bone formation was still in progress, the 45‐day group showed greater stability and bone consolidation. The PLLA/GO scaffolds appeared more integrated into the mineralized tissue, with less defined implant margins, indicating a more homogeneous incorporation into the surrounding bone. Regarding bone cells, osteocytes remained present in the lacunae of the matrix, as expected in more mature tissue. Osteoblasts were less evident but likely active on trabecular surfaces, contributing to the ongoing bone remodeling process.

Regarding the inflammatory response, we observed a reduction in the number of inflammatory cells surrounding the implant compared to the 15‐day group. The infiltration of macrophages and multinucleated giant cells, typical of a foreign body reaction, appeared less evident, suggesting a decline in acute inflammation and progression toward tissue adaptation (Figure 1E).

In the 60‐day group, the bone tissue surrounding the implant showed greater structural organization. The trabeculae were thicker and well‐formed, indicating that osteogenesis remained active and had reached a more stable phase compared to earlier stages. These characteristics suggest progressive bone maturation. The presence of osteocytes within the bone lacunae confirmed a more advanced stage of bone remodeling, while osteoblastic activity was less evident, possibly indicating that new bone deposition had reached a more stable stage.

The inflammatory response decreased significantly compared to earlier stages. The amount of connective tissue surrounding the implant decreased, suggesting good biocompatibility of the material, with a balanced and favorable tissue response for bone integration. The interface between the PLLA/GO scaffold and the bone tissue was less distinct, indicating that the implant was being incorporated more efficiently into the native bone tissue (Figure 1F).

**FIGURE 2:**
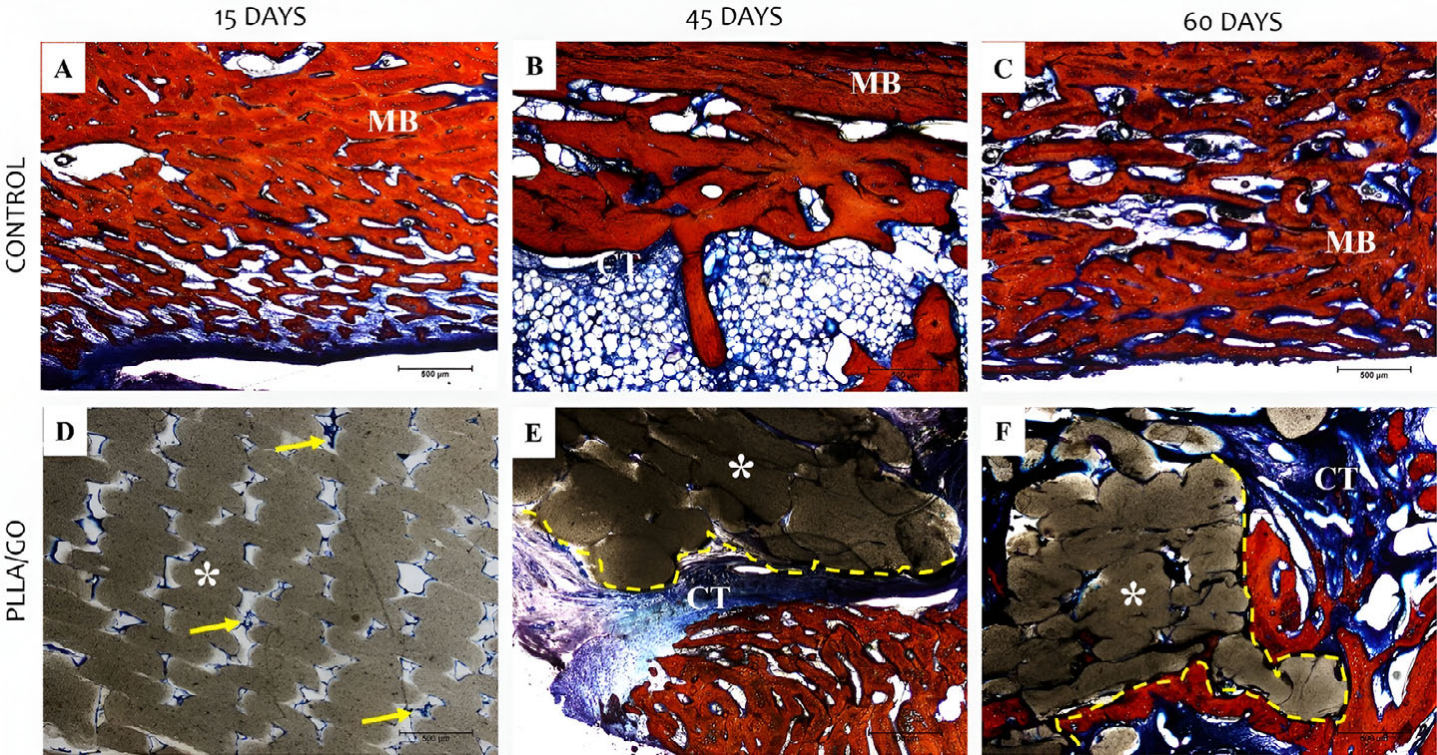
Histological images (Steven's blue staining) of goat mandibles from control (A–C) and PLLA/GO scaffold‐treated groups (D–F) at 15, 45, and 60 days. Mineralized matrix (MM, red), connective tissue (CT, blue), and scaffold regions () are shown. Yellow arrows indicate mineralized matrix deposition, and yellow dashed lines mark the PLLA/GO–matrix interface. Scale bar: 100 µm.

Steven's blue staining highlighted collagen components and mineralization, thus enabling the visualization of the mandibular bone structure in control tissues. The red staining marked the mineralized matrix, primarily composed of collagen and minerals such as calcium and phosphate in the form of hydroxyapatite crystals [37]. Blue staining appeared in the innermost regions (spaces between bone trabeculae), formed by loosely organized or less mineralized connective tissue, such as ossification zones (Figure 2A–C).

At 15 days, the dark portion (corresponding to PLLA/GO) remained largely intact, with unfilled spaces present and no clear evidence of osseointegration. Moreover, a thin blue layer surrounding the biomaterial indicated the formation of an organic matrix, suggesting that PLLA/GO provided a favorable environment for osteoconduction (Figure 2D).

At 45 days, the PLLA/GO‐mineralized tissue interface (blue and red areas) showed a gradual integration of the biomaterial, consistent with the expected behavior of osteoconductive materials, as described by Mendonça [38] and Tiberio et al. [39]. The presence of a mineralized matrix surrounding the biomaterial (blue area) suggests implant acceptance and the initiation of mineral deposition, with the surrounding cells contributing to matrix formation. Guo et al. [3] and Shuai et al. [40] reported that such biomaterials act as temporary scaffolds for the progressive deposition of bone matrix, which aligns with our findings. Moreover, cell‐like structures were observed within lacunae in the mineralized matrix (red area), suggesting ongoing bone tissue formation (Figure 2E).

At 60 days, mineralized tissue was evident around the biomaterial. Areas of newly formed matrix suggested active remodeling, with cells consistent with osteoblasts depositing new material along the biomaterial–tissue interface. Moreover, PLLA/GO was distributed irregularly within the tissue—a common response to biomaterials that promote ossification and may be partially or completely replaced by bone over time [41, 42, 43, 44]. As bone integrated with PLLA/GO, osteoblasts and other cellular elements infiltrated the biomaterial, creating a more disorganized and irregular interface due to the mixture of forming bone matrix and resorption areas (Figure 2F).

### 2.2 Quantitative Analysis of Mandibular Tissue Mineralization

**FIGURE 3:**
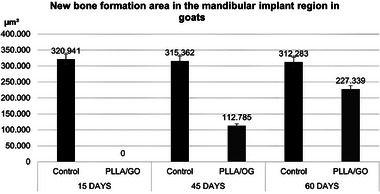
Quantification of the total area of new bone formation in control and PLLA/GO scaffold‐treated goat mandibles at 15, 45, and 60 days. Significant differences were observed between groups and time points.

The quantification of mandibular tissue mineralization areas is shown in Figure 3. At 15 days, the PLLA/GO group showed no detectable tissue formation (0 µm^2^) than the control group (320.941 µm^2^). This result may be attributed to the early phase of bone repair, which is dominated by inflammation and granulation tissue formation. These processes can delay bone matrix deposition, particularly in biomaterial implants that require an initial integration period, as suggested by Yu et al. [45].

At 45 days, the PLLA/GO group showed an increase in mineralized formation area (112.785 µm^2^), indicating progression in osteogenesis, while the control group maintained values similar to those observed earlier (315.362 µm^2^). This finding aligns with studies demonstrating that functionalized PLLA and GO scaffolds provide a favorable environment for osteoblast proliferation and differentiation, facilitating mineralized matrix deposition in later stages of bone healing [46, 47, 48].

Finally, at 60 days, tissue remodeling became evident, with an increase in mineralized area within the PLLA/GO scaffolds (227.339 µm^2^), while the control group showed a larger, yet comparable area (312.283 µm^2^). This increase suggests ongoing mineral deposition and potential integration of the scaffold with the surrounding tissue. Bioresorbable materials like PLLA and GO are gradually replaced by newly formed bone during remodeling, as described by Hu et al. [49] and Govindarajan et al. [50]. Our findings are consistent with the observations in those studies.

### 2.3 Immunohistochemical Profile of Goat Mandibles

Immunohistochemical staining for osteocalcin, a non‐collagenous protein secreted by osteoblasts, was used to assess osteoblast activity and the presence of bone matrix associated with mineralization. Additionally, VEGF labeling, a key regulator of angiogenesis, was employed to evaluate the progression of new bone formation and vascularization [51, 52].

**FIGURE 4:**
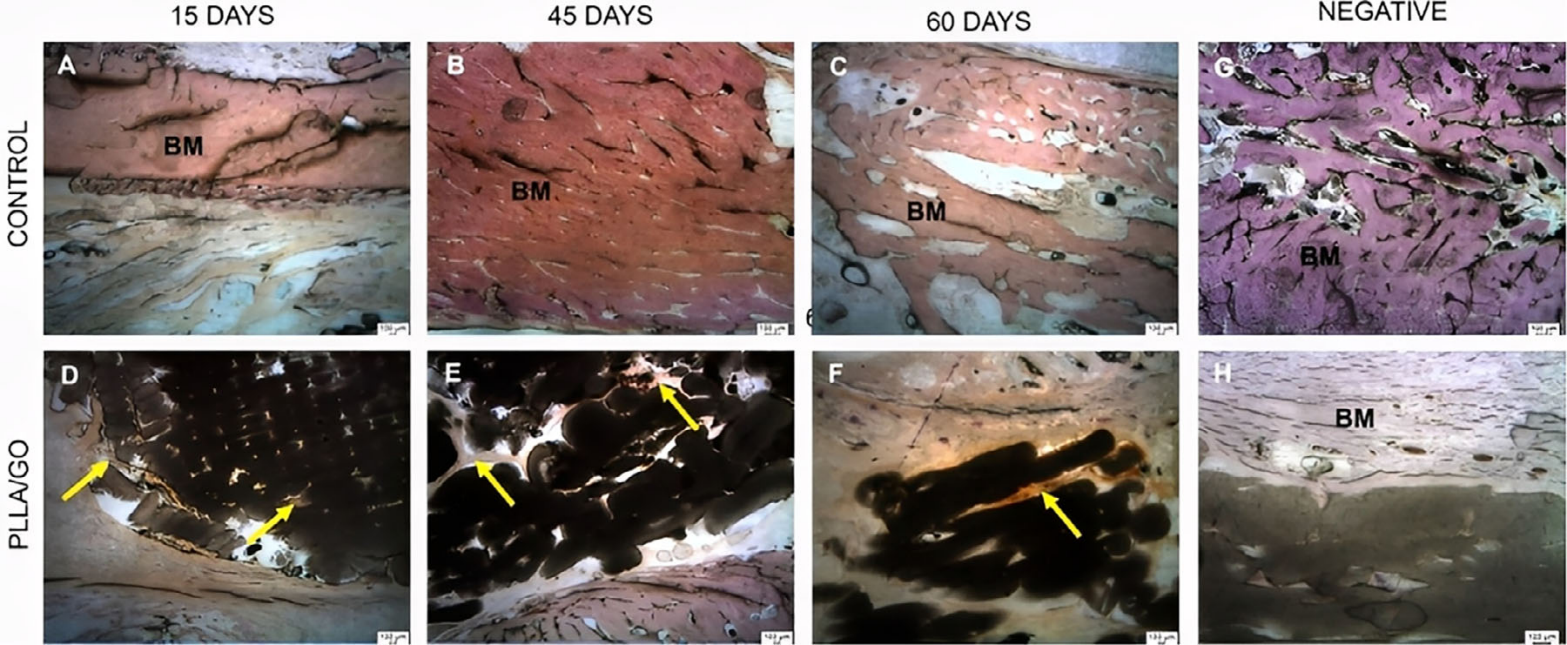
Immunohistochemical images of goat mandibles from control and PLLA/GO scaffold‐treated groups stained for osteocalcin at 15, 45, and 60 days (A–F). Osteocalcin‐positive staining is observed in osteocytes, within scaffolds (yellow arrows), and along the bone matrix (BM). Negative controls (G–H) correspond to tissue sections processed without the primary antibody. Scale bar: 100 µm.

Both the control and PLLA/GO groups exhibited positive osteocalcin staining at 15, 45, and 60 days (Figure 4A–F). Osteocalcin expression was detected in osteocytes within the scaffolds and along the mandibular bone matrix. Due to intense remodeling driven by functional and mechanical demands, osteocalcin staining highlighted regions of active and mature bone formation. This biomarker is essential for assessing the quality and stage of bone repair, particularly in studies involving biomaterial‐based scaffolds. [53]. Negative controls confirmed non‐reactivity for osteocalcin (Figure 4G,H).

The slides used for immunohistochemistry were prepared with acrylic support and embedded in resin, which resulted in more prominent traces and scratches that could be misinterpreted as technical flaws. However, this characteristic is inherent to the material. Unlike traditional glass slides used for paraffin‐embedded tissue sections, the mineralized nature of the mandibular tissue and scaffolds made paraffin embedding unfeasible. Therefore, we opted for resin embedding (methyl methacrylate), which better preserved bone structure and allowed for proper sectioning. Although the resin's composition can produce visual artifacts, it did not compromise the integrity of the results or the quality of the immunostaining.

**FIGURE 5:**
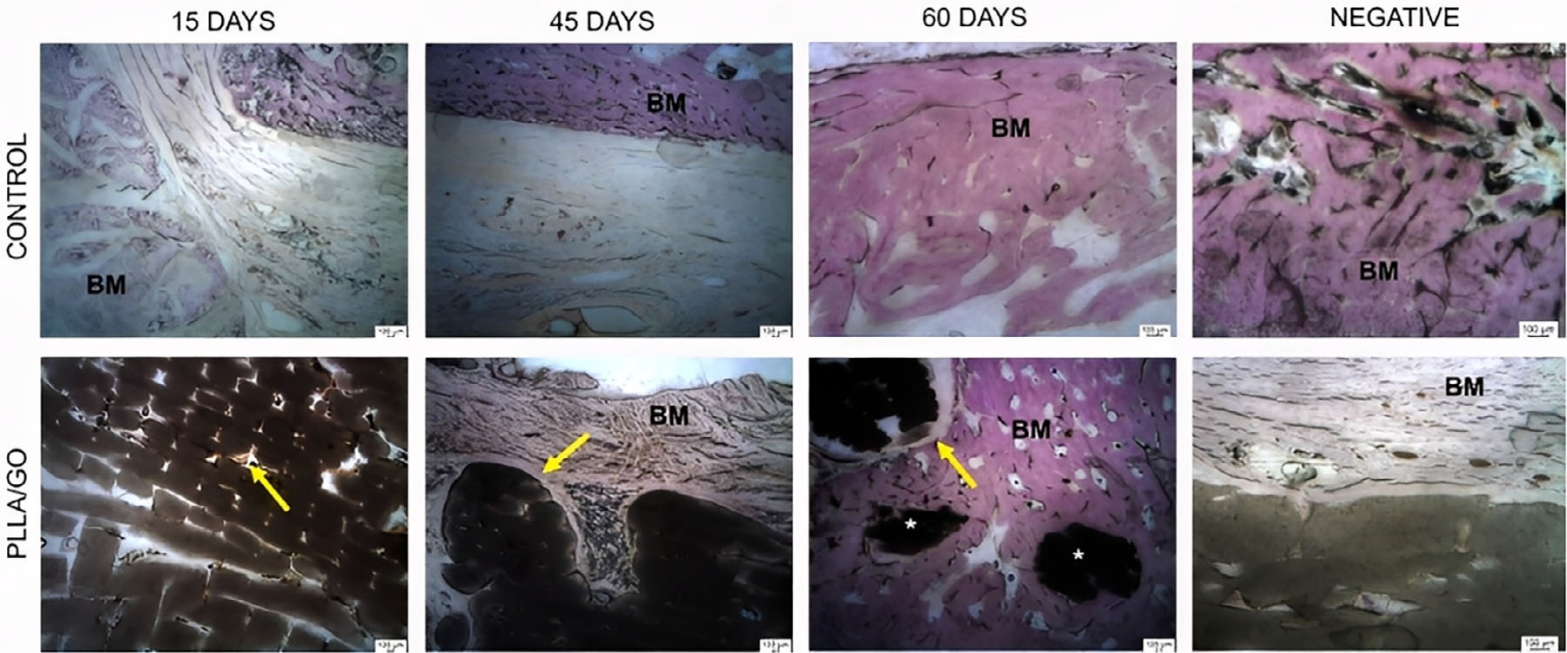
Immunohistochemical images of goat mandibles from control and PLLA/GO scaffold‐treated groups stained for VEGF at 15, 45, and 60 days (A–F). VEGF‐positive staining is indicated by yellow arrows, and biomaterial regions are marked with (*). Negative controls (G–H) were obtained from tissue sections incubated without the primary antibody. Scale bar: 100 µm.

In bone repair with biomaterials, VEGF is often used to assess the angiogenic response to implants [54, 55]. The positive VEGF staining observed in all samples (Figure 5A–F), in both the control and PLLA/GO groups at 15, 45, and 60 days, reinforces its crucial role in bone repair. The observed osteoblastic activity suggests that VEGF contributed to osteoblast differentiation and proliferation, which are essential for bone matrix deposition. The more intense VEGF staining around and within the PLLA/GO scaffolds at 45 and 60 days indicates both the biocompatibility of the biomaterial and its capacity to promote a robust angiogenic response. This vascularization is crucial for supplying nutrients and oxygen to the developing tissue, thus favoring osteogenesis [55, 56, 57].

The resorption observed at 60 days, along with an increase in bone tissue, indicates a synergistic interaction between the biomaterial and the biological microenvironment, facilitated by VEGF expression. These findings align with previous studies that identify VEGF as a key mediator of angiogenesis and osteogenesis during bone repair, particularly in biomaterial‐based models [58, 59, 60]. Therefore, the consistent VEGF expression throughout the experiment indicates that the scaffold not only supported but also actively enhanced bone regeneration by stimulating angiogenesis. Negative controls are shown in Figure 5G,H. Identical images were used in Figures 4G and 5G, and Figures 4H and 5H to depict the negative control.

## 3 Conclusion

Our results indicate that PLLA/GO has good biocompatibility with bone tissue. A remodeling response around the biomaterial was observed, characterized by areas of mineralized tissue formation at the biomaterial interface. This response suggests that PLLA/GO promotes osseointegration, gradually becoming incorporated into the developing bone tissue. At 60 days, a gradual decrease and irregularity in the structure of the PLLA/GO scaffold were evident, indicating biodegradation and resorption of the biomaterial. This result was further supported by the quantification of mineralized tissue areas, which showed a progressive increase over time. Immunohistochemical staining confirmed positive expression of osteocalcin and VEGF in both PLLA/GO and control scaffolds, indicating osteoblastic activity. Therefore, PLLA/GO scaffolds effectively support bone regeneration, gradually being replaced by natural bone tissue. These results demonstrate that PLLA/GO scaffolds promote bone regeneration and integration, supporting their potential for clinical application in mandibular repair.

## 4 Experimental Section

### Ethical Approval

This study was approved by the Ethics Committee for the Use of Animals at the Faculdade de Medicina Veterinária e Zootecnia, Universidade de São Paulo (protocol no. 9130071019) and the Universidade Estadual Paulista, Jaboticabal campus (protocol no. 2689/21).

### Animal Samples

Nine adult male goats (*Capra hircus*) were used and kept at the Central Bioterium of São Paulo State University (Jaboticabal/SP) until the conclusion of the study. Animal handling and feeding followed standard bioterium protocols. Each animal received two implants: a PLLA/GO scaffold with MSCs was implanted on the right antimere of the mandible, and an autologous bone fixed with a titanium plate was implanted on the left antimere as described by Santos‐Silva et al. [61]. Experimental groups were evaluated at 15 days (n = 3), 45 days (n = 3), and 60 days (n = 3).

### Material Composition and Fabrication of PLLA/GO Scaffolds

The production of the PLLA/GO scaffolds follow previously described protocols [6]. Briefly, graphene oxide (GO) was synthesized by chemical exfoliation of graphite (Nacional de Grafite Ltda, SP, Brazil) using a modified Hummers method [62]. PLLA (Evonik RESOMER L 210 S) was combined with 0.2% GO (w/w), and the mixture were extruded using a standard screw extruder to produce 1.75 mm filaments, which were then stored in a dehumidifier until 3D printing. Computed tomography (CT) scans (GE ACTS 16/32, GE Healthcare, Chicago, IL, USA) of the goat mandible were acquired and converted to STL files for planning the osteotomy and creating a critical mandibular defect. Subsequently, a STL file of the mandible was generated with the dimensions of the scaffold (4.5 cm × 3.0 cm) with a pore size of 0.43 mm. Osteotomy guides were modeled in Blender *software* (version 2.79b, Blender, Amsterdam, Netherlands) and printed using an FDM printer with ABS material, ensuring accurate scaffold placement during surgery.

### Histological Evaluation

**FIGURE 6:**
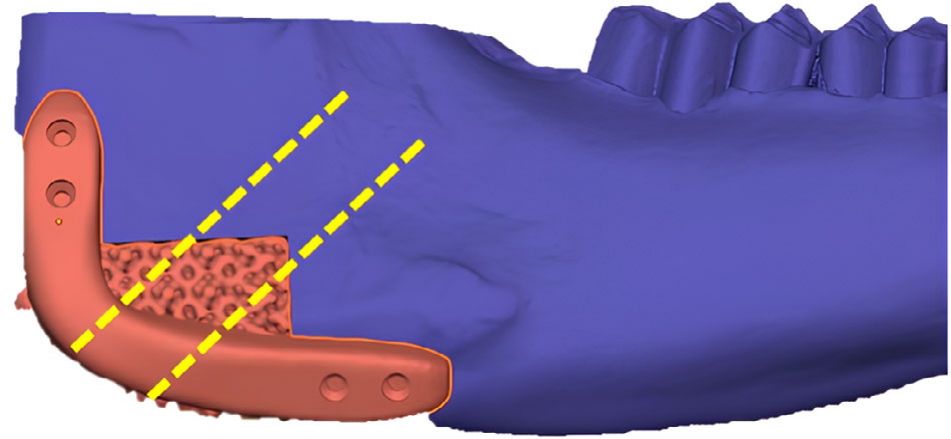
Schematic drawing showing the position (dashed line) where all histological sections (control and PLLA/GO scaffolds) were made in the goat mandible for microscopic analysis.

Control bone fragments (fixed with titanium plates) and PLLA/GO scaffolds were fixed in 4% paraformaldehyde for 48 h and then transferred to 70% ethanol. The samples were embedded in methyl methacrylate (C_5_H_8_O_2_, HL100.105, EXODO/HALOGENN) for 28 days, cut using a jeweler's saw, and sanded (200, 400, and 600 grit) until reaching a thickness of 5 µm. The slides were stained with hematoxylin and eosin (H&E) and Steven's Blue and analyzed using a light microscope (FV1000 Olympus IX91, Tokyo, Japan) at the Advanced Diagnostic Imaging Center of the Faculdade de Medicina Veterinária e Zootecnia, Universidade de São Paulo. Figure 6 illustrates the location and orientation of the histological sections. Observations regarding potential graft rejection due to persistent inflammatory responses were previously reported [6].

### Immunohistochemical Evaluation

Acrylic slides containing control bone tissue and PLLA/GO scaffolds were hydrated in two changes of 100% ethanol for 3 min each, followed by 95% and 80% ethanol for 1 min each. The slides were then rinsed in distilled water. Next, they were incubated with a pepsin working solution for 15 min at 37 °C in a humidified chamber. After cooling to room temperature for 10 min, the samples were washed twice with PBS‐Tween 20 for 2 min each. Endogenous peroxidase activity was blocked using 3% hydrogen peroxide in distilled water for 30 min in the dark.

The primary antibodies used were Osteocalcin (NB100‐62801, Interprise USA Corporation) and VEGF (NB100‐648, Interprise USA Corporation). Overnight incubation was performed at 4 °C in a humidified chamber. After three washes (2 min each), the slides were incubated with an anti‐mouse IgG secondary antibody (NB7539, Interprise USA Corporation) for 1 h. Antibody details are shown in Table 1. The reaction was developed using DAB (DABS‐999, Substrate Spring, BIOGEN), following the manufacturer's instructions, and counterstained with hematoxylin for 15 s. Finally, the slides were photographed and examined under a light microscope (NIKON Eclipse 80I, Nikon Instruments Inc., Melville, NY, USA).

**TABLE 1:** List of primary antibodies used for immunohistochemical analyses of PLLA/GO scaffolds and controls in goat mandibles.

Primary antibody	Supplier	Cat. no.	Antigen retrieval	Dilution	DAB
ECM markers					
Osteocalcin	Novus Biological	NB100‐62801	PIER	1:100	Spring Bioscience Kit
Vascularization markers					
VEGF	Novus Biological	NB100‐648	PIER	1:200	Spring Bioscience Kit

### Quantification of New Bone Formation

**FIGURE 7:**
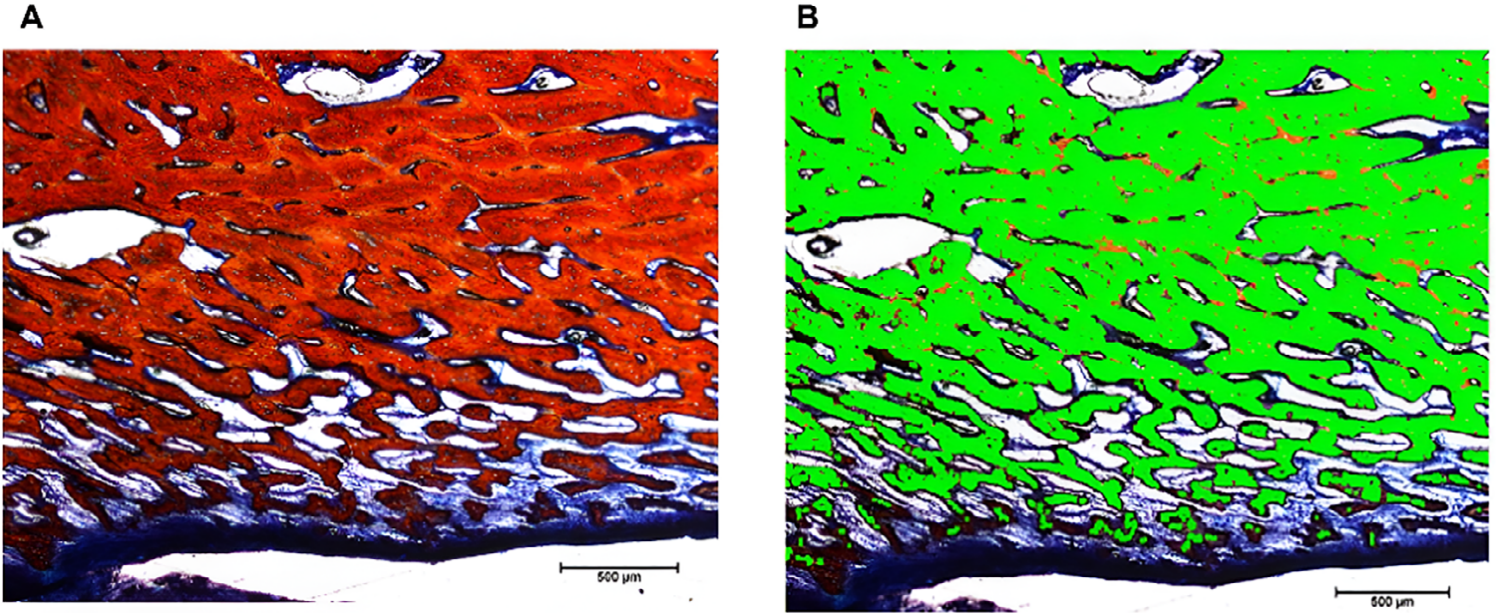
Standardization of images in ImageJ software. (A) Original image; (B) Segmentation of mineralized tissue (green).

**FIGURE 8:**
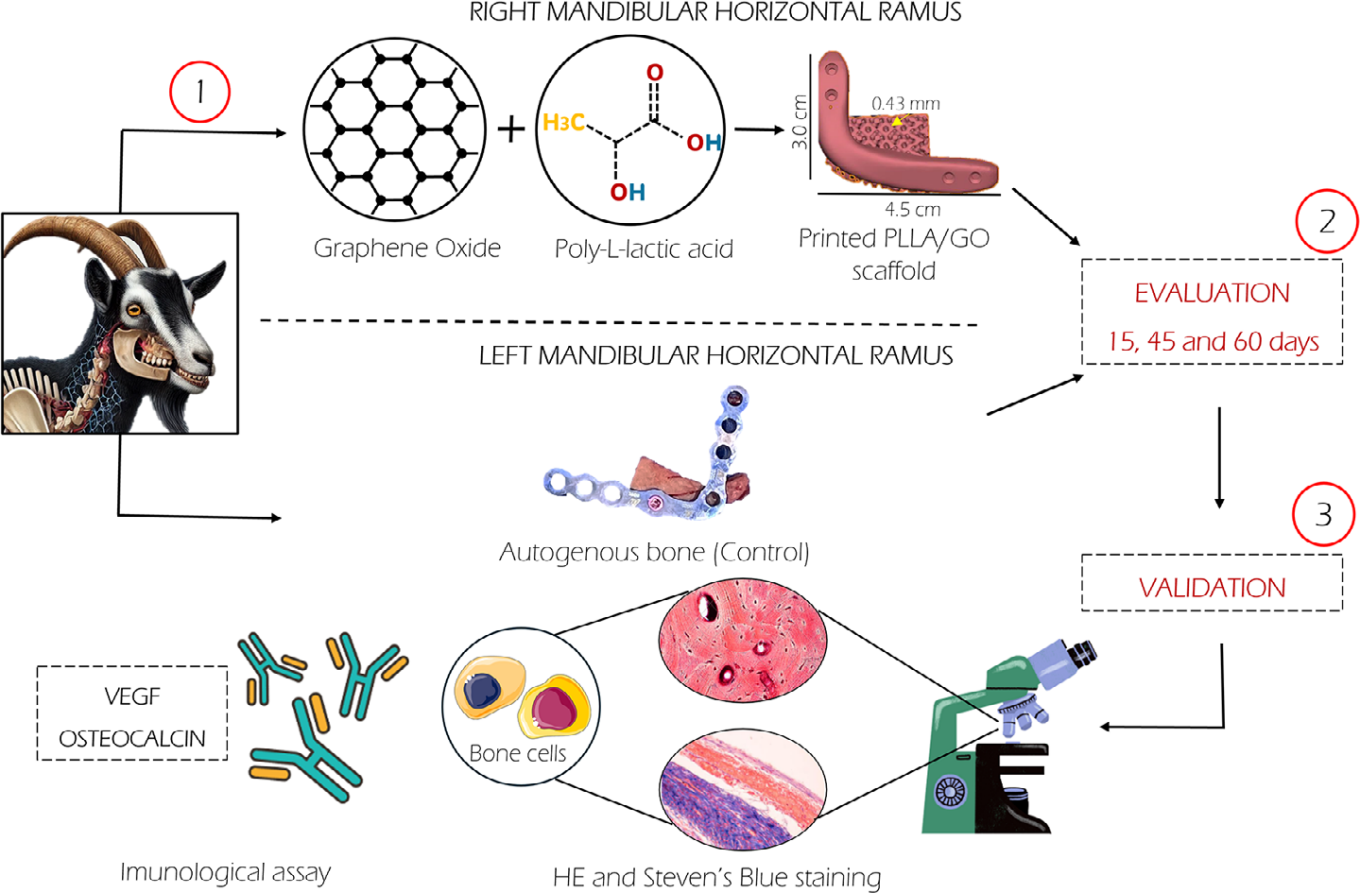
Schematic summary of the methodology. (I) Association of PLLA and GO for 3D printing of the PLLA/GO scaffold (right horizontal ramus) and the control, which consisted of a titanium plate fixation (left horizontal ramus); (II) Animals were evaluated at 15, 45, and 60 days; (III) Validation was performed through histological and immunohistochemical assays to assess tissue integration and repair at the bone‐implant interface.

To evaluate the presence of mineralized tissue, three images of the right and left antimeres from each animal were acquired following histological processing and staining with Steven's Blue. The areas of interest were defined based on the visibly mineralized regions in the histological sections. These regions were manually selected within the defect or implant area, and their corresponding area was calculated using ImageJ software in square micrometers (µm^2^). The term “mineralized tissue” was used to describe these areas, as Steven's Blue staining allows the identification of mineralized tissue but does not enable precise distinction between immature and mature bone, (Figure 7). These images were analyzed using ImageJ win64 (version 1.48). We then estimated the mean area of the implant regions (in µm) as follows. First, the image was opened in the software. Second, the scale was calibrated by selecting the straight line tool and measuring 500 µm. Third, the Analyze/Set Scale option was selected, and the actual distance (500 µm) was defined. Fourth, the image was converted to an 8‐bit format using Image/Type/8‐bit. Fifth, the red‐stained area was segmented by selecting Image/Adjust/Color Threshold and adjusting the values to isolate only the red regions. The selection was confirmed using the Apply option. Lastly, the area was measured by selecting Analyze/Set Measurements/Area, followed by Analyze/Measure to obtain the area in µm^2^. Data were tabulated in Microsoft Excel and analyzed using GraphPad Prism 7.00 (version 9.5.1). A two‐way ANOVA test was performed with a significance level of 0.05. Figure 8 summarizes the methodological steps, from scaffold printing to post‐surgery analyses.

## Funding

The São Paulo Research Foundation (FAPESP, grant number 2021/05445–7) and the Coordination for the Improvement of Higher Education Personnel (CAPES, grant number 88887.488264/2020‐00).

## Conflicts of Interest

The authors declare no conflicts of interest.

## Data Availability

The data that support the findings of this study are available from the corresponding author upon reasonable request.
